# Good Health and Wellness in Indian Country: A New Partnership and Approach

**DOI:** 10.5888/pcd16.190214

**Published:** 2019-08-15

**Authors:** Ursula E. Bauer, David K. Espey

**Affiliations:** 1 Office of the Deputy Director for Non-Infectious Diseases, Centers for Disease Control and Prevention, Atlanta, Georgia; 2 National Center for Chronic Disease Prevention and Health Promotion, Centers for Disease Control and Prevention, Atlanta, Georgia

The health and wellness of American Indians and Alaska Natives has steadily improved since the dark days of the mid-twentieth century, when population levels reached an all-time low and termination of sovereign status threatened tribal existence (1). During the past 50 years, the federal government’s extension of basic rights, such as the free exercise of religion, and activism, court victories, and deployment of tools for economic development by American Indians and Alaska Natives have ushered in a period of growth and resurgence. As sovereign peoples, however, American Indians and Alaska Natives have not escaped broader societal trends in health and disease, such as the epidemics of alcohol and tobacco use, the obesity epidemic that emerged in the late 1980s and 1990s and was followed by increases in rates of type 2 diabetes, and the challenges of opioid and other substance misuse that have plagued the country in the 20th and 21st centuries.

The Centers for Disease Control and Prevention’s (CDC’s) Good Health and Wellness in Indian Country (GHWIC) seeks to build on a new sense of possibility in Indian Country. This new sense of possibility is producing Native-owned businesses, developing and marketing Native cuisine, supplying traditional Native foods to government food distribution programs, expanding the number of Native language speakers through immersion programs, and, in so many other ways, lifting up vibrant, persistent, and resilient cultures. As Andrade et al describe in this collection of articles on Indian wellness in *Preventing Chronic Disease*, the GHWIC program strives to incorporate “tribal wisdom to protect and promote physical, mental, and spiritual wellbeing; . . . [to] work upstream addressing drivers of poor health through culturally appropriate practices; and [to solve] the problem of funding only a small number of tribes to do disease-specific work in favor of a holistic approach that reaches deeply and widely into Indian Country” (2). Over time and with additional investment by Congress, the initial GHWIC cooperative agreement grew into a portfolio of programs that addresses public health gaps, unleashes the power of traditional practices, and deploys a holistic approach to chronic disease prevention.

As anyone who seeks to build something permanent, useful, and relevant knows, the first step is to build community. This holds for public health programs as much as for community centers, neighborhood health clinics, and grocery stores in a food desert. If the community is not together, if it is not functioning cohesively, if it does not possess a shared vision and sense of possibility, whatever structure or program is being built will struggle in its purpose to provide possibility, bring the community together, and address a community need (3). Thus, a first step for GHWIC was to bring the GHWIC grantees together and undertake the hard work of community building. As described by Williams et al, GHWIC embarked on this process by establishing a community of practice and enlisting the support of the University of New Mexico Extension for Community Health Outcomes (ECHO) platform and approach. “ECHO combines evidence-based education, workforce development, and collaborative problem solving to increase practitioners’ capacity in specialty areas. The model uses videoconferencing and subject matter expertise to facilitate case-based learning among practitioners and to share best practices” (4). Although the ECHO model was originally established to support health care delivery, GHWIC adapted it to support a community of practice that democratizes access to and disseminates peer solutions in service of chronic disease prevention and health promotion in Indian Country.

With community building underway, progress could be made toward two essential goals of GHWIC: to reach *deeply* into Indian Country and to reach *widely* into Indian Country. As described by Andrade et al, the GHWIC program sought to address critical weaknesses in previous CDC work in Indian Country: the problem of so many federally recognized tribes and insufficient human and financial resources to support them all (2). One of the many innovations of GHWIC is funding individual tribal nations directly and funding tribal organizations with area-wide reach to serve and support most or all of the tribes or villages in their service areas. Two articles in this collection showcase this approach. Alonso et al describe the rich array of interventions implemented by the Winnebago Tribe of Nebraska, driven by a community assessment and with the engaged support of tribal government and tribal members to institutionalize supports for healthy behaviors (5). With direct funding, the grantee made extraordinary progress on a diverse range of health improvement strategies. Redwood et al describe an ambitious approach to serving Alaska Natives that reaches across a large area and many villages. Using existing networks, the Alaska Native Tribal Health Consortium provides knowledge, tools, and resources to network members who implement policies, systems, and environmental improvements across the state that, to date, have supported the health and wellness of 46,000 Alaska Natives (6). These grantees are just two examples of how the GHWIC program reaches deeply and widely, through a dual funding model, into Indian Country.

No CDC program is complete without robust evaluation. For GHWIC, evaluation is being conducted at 3 levels: the individual grantee, the area, and across Indian Country. Each grantee is responsible for demonstrating the impact of its work by collecting performance measures on access to healthy and traditional foods and their preparation; practices and policies to increase physical activity; and development, implementation, or enhancement of commercial tobacco-free policies. Grantees also developed success stories that qualitatively and visually highlight their activities or programs. Tribal Epidemiology Centers (TECs) in each administrative area of the Indian Health Service support area-wide evaluations. These evaluations demonstrate progress toward health improvements and include regional briefs and policy briefs that summarize changes that have occurred across the area as a result of GHWIC funding and support. The Urban Indian Health Institute conducts the program-wide or nations-wide evaluation, demonstrating the impact of the cooperative agreement on progress toward health outcomes in Indian Country as a whole. As Lawrence and James explain, the evaluation framework draws on indigenous approaches and recognizes the importance of culture to American Indian and Alaska Native health, of local context and community knowledge in documenting program progress, and of locally tailored metrics to ensure adherence to tribal protocols and cultural priorities (7). With the first 5 years of the GHWIC program set to close in September 2019, Lawrence and James present evaluation findings from the first 3 years of the program and share important examples of the increase in opportunities for healthy behaviors, drawn from traditional tribal practices, that GHWIC supported among grantees and across Indian Country. For example, as a result of GHWIC efforts during 2014–2017, approximately 15,000 American Indians and Alaska Natives in 16 tribal or village settings benefited from low-sodium nutrition guidelines and 77 new tribal settings promoted healthier food (7). Similarly, during the same period, more than 14,500 American Indians and Alaska Natives increased access to physical activity through GHWIC and 91 new policies that promote physical activity (7). Further evaluation is being conducted by other CDC programs. Although the GHWIC program is still underway as these articles go to press, they document early outcomes and the promise of future strong results for the GHWIC approach.

When the inaugural GHWIC program was launched in 2014, tribal health leaders serving on CDC’s Tribal Advisory Committee asked the agency to learn about tribal practices used by Indian people to keep their communities healthy and well. A second article by Andrade et al describes the process by which CDC embarked on this assignment and the exciting Tribal Practices for Wellness in Indian Country program, launched in 2018, that resulted from listening, learning, engaging, and understanding (8). Thanks to years of collaboration with the Tribal Advisory Committee and tribes, CDC was ready to deploy new Congressional funding to support the Tribal Practices grant program. Between the launch of the GHWIC program in 2014 and the Tribal Practices program in 2018, CDC was able, again with generous support from Congress, to robustly support TECs to build their public health authorities and increase their capacity to serve the public health needs of tribes in their service areas. Although TECs have been well-integrated into the work of GHWIC from the initial planning of the program, funding had lagged and TECs were stretched until additional resources became available in 2017 to support their critical work.

With the addition of the Tribal Epidemiology Centers Public Health Infrastructure program and Tribal Practices for Wellness in Indian Country program to the initial GHWIC program, the Good Health and Wellness in Indian Country portfolio now supports individual tribes, tribal organizations, urban Indian centers, and TECs to prevent chronic disease and promote health in ways that reflect indigenous culture and heritage, build on American Indian and Alaska Native strengths and resiliency, and implement what tribes and villages know will keep their people healthy and well. The collection of articles in *Preventing Chronic Disease* chronicles the GHWIC journey, from conception to behavioral outcomes, as new programs launch and the first 5 years of the GHWIC program come to a close. Together, the programs provide a model for public health practice across the country and around the world that seeks to be relevant to the people being served, to uplift culture and respect local knowledge, and to institutionalize sustainable health improvements.
